# Urinary Levels of High Mobility Group Box-1 Are Associated with Disease Activity in Antineutrophil Cytoplasmic Autoantibody-Associated Vasculitis

**DOI:** 10.1371/journal.pone.0123586

**Published:** 2015-04-17

**Authors:** Tian-Tian Ma, Huan Wang, Chen Wang, Dong-Yuan Chang, Ming-Hui Zhao, Min Chen

**Affiliations:** Renal Division, Department of Medicine, Peking University First Hospital, Beijing, China; University of Leicester, UNITED KINGDOM

## Abstract

**Background:**

High mobility group box-1 (HMGB1), a kind of pro-inflammatory mediator, is associated with inflammatory conditions and tissue damage. Our previous study demonstrated that the circulating levels of HMGB1 correlated with disease activity of antineutrophil cytoplasmic antibody (ANCA)-associated vasculitis (AAV). In the current study, we aimed to measure urinary levels of HMGB1 in AAV patients, correlated them to clinical activity index and analysed the immunohistochemical HMGB1 staining in kidney specimens.

**Methods:**

50 patients with AAV in active stage and 56 patients with AAV in remission were recruited. The urinary levels of HMGB1 were determined by enzyme-linked immunosorbent assay. Moreover, renal biopsy specimens from 27 patients with active AAV were randomly collected to evaluate the deposition of HMGB1.

**Results:**

Urinary HMGB1 levels in AAV patients in active stage were significantly higher than those in AAV patients in remission and healthy controls (1.46 [0.56-3.43] versus 0.38 [0.10-1.35] mg/μmolCr, P=0.001; 1.46 [0.56-3.43] versus 0.48 [0.40-0.60] mg/μmolCr, P=0.000, respectively). Further analysis found that urinary levels of HMGB1 correlated with erythrocyte sedimentation rate (r=0.354, p=0.012), C-reactive protein (r=0.289, p=0.042), and Birmingham Vasculitis Activity Score (r=0.350, p=0.013). Renal tissue of active AAV patients showed HMGB1 was mainly expressed in the cytoplasm and the extracellular space. The percentage of HMGB1-negative nuclei in renal tissue of patients with active AAV was significantly higher than that in normal controls (60.6±20.2 % versus 2.7±0.6 %, p<0.01).

**Conclusion:**

Urinary levels of HMGB1 may be associated with the disease activity in AAV patients.

## Introduction

Antineutrophil cytoplasmic antibody (ANCA)-associated vasculitis (AAV) is characterized by a necrotizing inflammation of the small vessels. AAV includes microscopic polyangiitis (MPA), granulomatosis with polyangiitis (GPA) and eosinophilic granulomatosis with polyangiitis (EGPA) [1]. The kidney is one of the most commonly affected vital organs in AAV, characterized by pauci-immune focal necrotizing crescentic glomerulonephritis [2].

High mobility group box 1 (HMGB1) is a 30-kD highly conserved DNA-binding nuclear protein. It can not only be actively released from macrophages and monocytes by several proinflammatory stimuli, but also be passively released from necrotic cells [3]. Through binding to its receptors, including receptor of advanced glycation end products (RAGE), toll-like receptor (TLR)-2, TLR-4, and the intracellular receptor TLR-9 [4–6], HMGB1 performs the role of a proinflammatory mediator, such as stimulating monocytes to secrete a specific subset of proinflammatory cytokines, including tumor necrosis factor-α (TNF-α) and interleukin-1 (IL-1) [7].

It has been demonstrated that HMGB1 plays an important role in the pathogenesis of several autoimmune diseases, including rheumatoid arthritis (RA), inflammatory bowel disease (IBD) and systemic lupus erythematosus (SLE) [8–10]. In recent years, the role of HMGB1 in the pathogenesis of AAV is getting recognized. Henes et al found that serum levels of HMGB1 correlated with the burden of granulomatous inflammation in GPA [11]. Our previous study demonstrated that the circulating levels of HMGB1 correlated with disease activity of AAV [12], although it remains controversial in some other studies [13]. Bruchfeld et al found serum levels of HMGB1 are higher in AAV patients with renal lesions [14]. However, the clinical and pathological significance of urinary levels of HMGB1 in AAV patients with renal lesion is not clear yet. In the current study, we measured urinary levels of HMGB1 in AAV patients, correlated them to clinical activity index and analysed the immunohistochemical HMGB1 staining in kidney specimens.

## Patients and Methods

### Patients and samples

Fifty patients with active AAV diagnosed in Renal Division, Peking University First Hospital between May 2008 and December 2013 were recruited in this study. All these patients had renal involvement of AAV. The research was in compliance of the Declaration of Helsinki and approved by the ethics committee of the Peking University First Hospital. Written informed consent was obtained from each participant as early as the first intervention, e.g., renal biopsy. Urine samples from these patients were collected before the initiation of immunosuppressive treatment. Forty-seven of the 50 patients received renal biopsy at diagnosis and before the initiation of immunosuppressive treatment. Urine samples of 56 patients with AAV, who achieved complete remission after immunosuppressive therapy, were also collected at their regular ambulatory visits. Among the above-mentioned AAV patients, there were 19 patients who had sequential urine samples, i.e., both in active stage and remission.

Twenty-seven paraffin-embedded sections of renal biopsy specimens were randomly collected from above-mentioned 47 patients who received renal biopsy to evaluate the deposition of HMGB1. Renal tissues from two unaffected parts of the kidneys of patients with renal cell carcinoma were used as controls. Ethics approval regarding these two patients was obtained separately from the ethics committee of our hospital.

All these patients met the Chapel Hill Consensus Conference criteria for AAV [1]. Patients with secondary vasculitis or coexistence of other renal disease were excluded. Disease activity was assessed in accordance with the Birmingham Vasculitis Activity Score (BVAS) [15]. “Remission” was defined as “absence of disease activity attributable to active disease qualified by the need for ongoing stable maintenance immunosuppressive therapy” (complete remission), or “at least 50% reduction of disease activity score and absence of new manifestations” (partial remission), as described previously [16]. All these patients were regularly followed up in the out-patient clinics specific for ANCA-associated vasculitis of our hospital at regular intervals of about 1 month during the induction therapy, and about 3 months during the maintenance therapy, or whenever clinically indicated.

Urine samples of 13 patients with biopsy-proven lupus nephritis in active stage were collected as the disease control. All these patients fulfilled the 1997 American College of Rheumatology revised criteria for systemic lupus erythematosus [17]. Urine samples of 17 age- and sex-matched healthy blood donors were collected as the normal control. The urine was stored in aliquots at -80°C until use. When testing, after rapid thawing at 37°C, the frozen specimens were transferred immediately onto ice before use within 1 hour. Repeated freeze/thaw cycles were avoided.

### Detection of serum ANCA

ANCA tests were performed by both indirect immunofluorescence assay and antigen-specific enzyme-linked immunosorbent assay, according to the manufacturer (Euroimmun, Lübeck, Germany). In indirect immunofluorescence assay, cytoplamic ANCA (cANCA) and perinuclear ANCA (pANCA) were distinguished. In antigen-specific enzyme-linked immunosorbent assay, two highly purified known ANCA antigens, proteinase 3 (PR3) and myeloperoxidase (MPO) were used as solid-phase ligands.

### Measurement of urinary HMGB1

The urine samples of patients and controls were concentrated 60 times using Vivaspin (Millipore Corporation, Massachusetts, USA). Urinary levels of HMGB1 were tested using commercially available ELISA kits (Shino-TEST). The assay was conducted according to the manufacturer’s instructions. Urinary HMGB1 was expressed as HMGB1/Cr ratio (mg/μmolCr) to correct for differences in dilution.

### Renal histology

Renal histology of patients with AAV was evaluated according to the previous standardized protocol [18–19]. The presence of glomerular lesions, including fibrinoid necrosis, crescents, and glomerulosclerosis were calculated as the percentage of the total number of glomeruli in biopsy findings. Interstitial and tubular lesions were scored semiquantitatively on the basis of the percentage of the tubulointerstitial compartment that was affected per the following: interstitial infiltrate (“-” for 0%, “+” for 0–20%, “++” for 20–50%, and “+++” for >50%), interstitial fibrosis (“-” for 0%, “+” for 0–50%, and “++” for >50%), and tubular atrophy (“-” for 0%, “+” for 0–50%, and “++” for >50%). Furthermore, according to the histopathologic classification proposed by Berden et al, patients were classified as focal, crescentic, mixed and sclerotic ANCA-associated glomerulonephritis [20].

### Immunohistochemical staining of HMGB1 in renal biopsies

Immunohistochemical staining of HMGB1 in renal biopsies was performed as described previously [21]. In brief, kidney sections (4 μm) were used for all staining experiments. Sections were deparaffinised. Next, endogenous peroxidase blocking and antigen retrieval was performed. Slides were incubated with rabbit anti-HMGB1 antibody (Abcam, Cambridge, UK). Subsequently, slides were incubated with HRP-labeled secondary antibodies (DakoCytomation, Glostrup, Denmark). Next, slides were counterstained with hematoxylin. The renal staining of HMGB1 was evaluated by the Image Pro Plus analysis software 6.0 (Media Cybernetics, Silver Spring, MD). Cellular distribution of HMGB1 was determined in the kidney by counting one hundred nuclei in three brightfield pictures and scoring both HMGB1-positive (brown) and HMGB1-negative (blue) nuclei. Results are expressed as the percentage of negative cells. In each assay, a primary isotype Ig control has been used as the negative control.

### Statistical analysis

Data were expressed as mean±SD (for data that were normally distributed), or median and interquartile range (IQR; for data that were not normally distributed). Differences of quantitative parameters between groups were assessed using the *t*-test (for data that were normally distributed) or the non-parametric test (for data that were not normally distributed). Differences of qualitative results were compared using the chi-square test. Pearson’s test or Spearman’s test was used for correlation analysis as appropriate. Given that the results of urinary HMGB1 were not normally distributed, by doing logarithmic transformation we converted the original data to its normal distribution representation for such adjustment. A *P* value less than 0.05 was considered to be statistically significant. All the statistics were analyzed using SPSS statistical software (version 13.0, Chicago, Illinois, USA).

## Results

### Demographic and general data

Among the 50 patients with AAV in active stage, 23 patients were male, and 27 patients were famale, with an age of 60.6±14.7 (range 23–83) years at diagnosis. Forty-one patients were diagnosed as MPA while the other 9 were diagnosed as GPA. The level of BVAS was 23.0±6.9 (range 8–44). The level of initial serum creatinine was 340.1±239.9 (range 51.3–962.0) μmol/L. The clinical and histopathological data were listed in Table 1.

**Table 1:** Clinical and histopathologic data of patients with AAV on diagnosis.

	Values
Total number of patients	50
Gender (male/female)	23/27
Age at diagnosis of diaease (years, mean±sd)	60.6±14.7
Initial Scr(μmol/L, mean±sd)	340.1±239.9
Urinary protein (g/24 hr, median,IQR)	1.3(0–7.5)
MPO-ANCA/PR3-ANCA	46/5
Skin rash	1
Arthralgia	1
Muscle pain	2
Pulmonary involvement	15
ENT involvement	11
Ophthalmic involvement	5
Nervous system involvement	1
Gastrointestinal involvement	0
BVAS(mean±sd)	23.0±6.9
Renal biopsy	47
Total crescents (mean±sd)	12.7±10.3
Cellular crescents(mean±sd)	11.3±9.7
Fibrous crescents median(IQR)	0(0–17)
Interstitial infiltration (-/+/++/+++)	1/4/22/20
Interstitial fibrosis (-/+/++)	6/11/30
Tubular atrophy (-/+/++)	5/24/18

[Abbreviations]: AAV, anti-neutrophil cytoplasmic antibody–associated vasculitis; ANCA, antineutrophil cytoplasmic antibodies; BVAS, Birmingham Vasculitis Activity Scores; ENT, ear, nose, and throat; IQR, interquartile range; MPO, myeloperoxidase antibodies; PR3, proteinase 3 antibodies; s.d., standard deviation; Scr, serum creatinine.

Among the 56 patients with AAV in remission, 24 patients were male and 32 were female. The level of serum creatinine at sampling was 161.3±103.4 (range 66.0–459.0) μmol/L. Regarding the BVAS levels of these 56 patients at remission, 54 patients were 0; one patient was 1 and the other one patient was 2, respectively.

### Urinary HMGB1 levels

**Fig 1:**
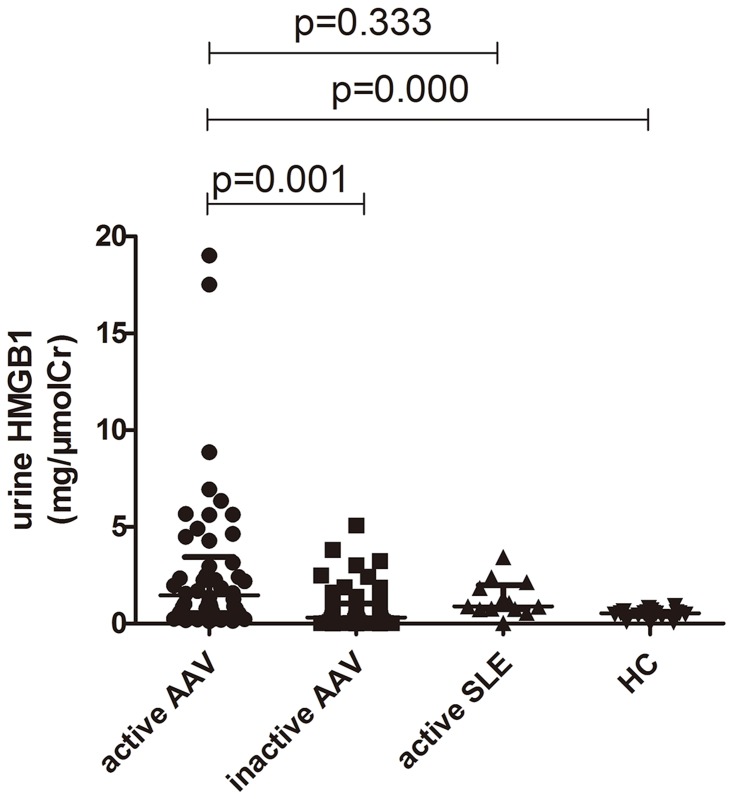
Urinary levels of HMGB1 in AAV patients in active and remission stage. SLE: systemic lupus erythematosus; HC: healthy controls.

Urinary levels of HMGB1 were normalized for urinary creatinine levels to correct for differences in dilution. The urinary HMGB1 levels in AAV patients in active stage were significantly higher, compared with AAV patients in remission and healthy controls (1.46 [0.56–3.43] versus 0.38 [0.10–1.35] (mg/μmolCr), P = 0.001; 1.46 [0.56–3.43] versus 0.48 [0.40–0.60] (mg/μmolCr), P = 0.000, respectively). ROC analysis was also used to assess the validity of urinary HMGB1 levels in identifying disease activity, AUC was 0.748 (95%CI 0.652–0.843), P = 0.000. No significant difference was found in urinary HMGB1 levels between AAV patients in active stage and patients with lupus nephritis (1.46 [0.56–3.43] versus 0.88 [0.75–1.98] (mg/μmolCr), P = 0.333) (Fig 1).

**Fig 2:**
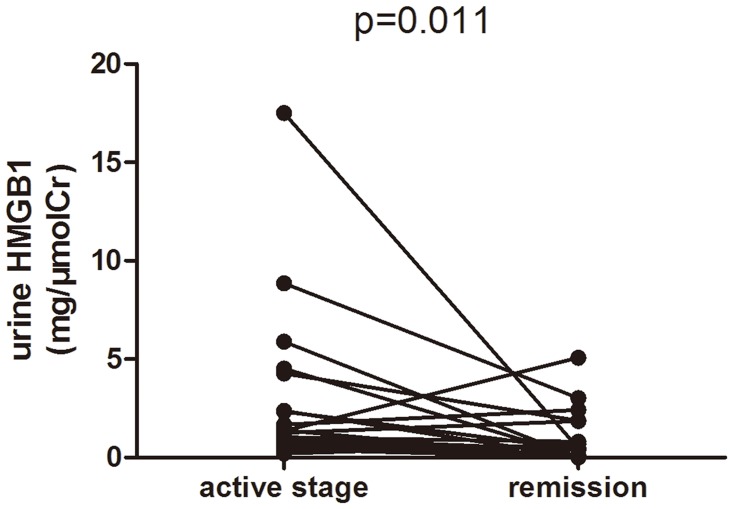
Changes of urinary HMGB1 levels in 19 AAV patients with sequential urinary samples.

Urinary levels of HMGB1 in nineteen AAV patients with paired urinary samples of both active stage and remission were then compared. The urinary levels of HMGB1 were significantly lower in remission than those in active stage (median 1.38 [0.89–4.47] versus 0.40 [0.23–1.87] (mg/μmolCr), P = 0.011). Sixteen out of these nineteen patients had a decrease in urinary level of HMGB1 in remission compared with those in active stage. Only three patients had higher urinary levels of HMGB1 than in active stage; all these three patients were in complete remission, with BVAS of zero (Fig 2).

### Association between urinary HMGB1 levels and clinicopathological parameters of patients with active AAV

**Fig 3:**
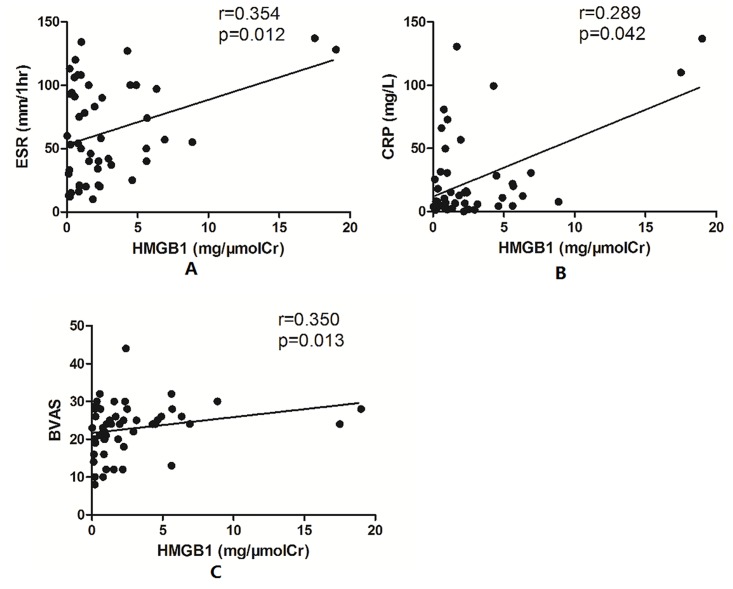
Urinary levels of HMGB1 correlated with ESR, CRP, and BVAS. A: Association between urinary levels of HMGB1 and ESR. B: Association between urinary levels of HMGB1 and CRP. C: Association between urinary levels of HMGB1 and BVAS.

The urinary levels of HMGB1 correlated with erythrocyte sedimentation rate (ESR) (r = 0.354, p = 0.012), C-reactive protein (CRP) (r = 0.289, p = 0.042), and Birmingham Vasculitis Activity Score (BVAS) (r = 0.350, p = 0.013) (Fig 3). However, no significant association was found between urinary HMGB1 levels and the renal histological parameters, and no significant difference was found in urinary HMGB1 levels among the four histopathological subtypes, i.e. focal, mixed, crescentic, or sclerotic categorization.

### Release of nuclear HMGB1 in the kidney

**Fig 4:**
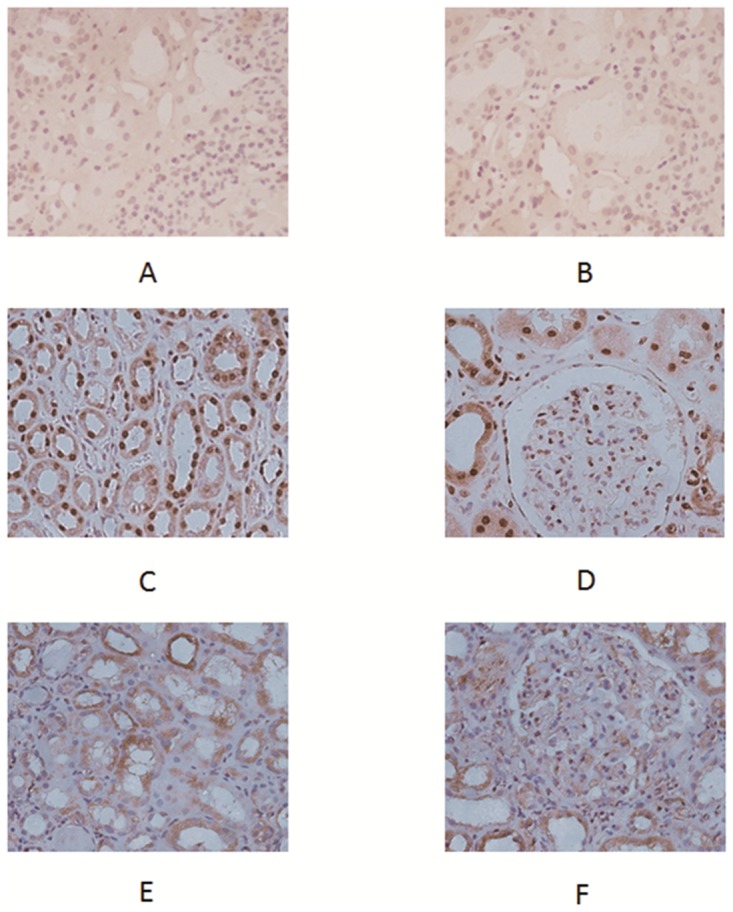
Expression of HMGB1 in renal tissue of active AAV patients and controls. A and B showed isotype Ig staining in control renal tissue. Biopsy taken from normal renal tissue showed expression of HMGB1 mainly inside nuclei (C, D). Renal tissue of the active AAV patient showed strong cytoplasmic and extracellular staining for HMGB1 in tubuli and glomeruli (E, F).

In normal renal tissues, HMGB1 was mainly present in the nuclei. However, in biopsy sections from patients with AAV in active stage, HMGB1 was mainly expressed in the cytoplasm and the extracellular space (Fig 4). The percentage of HMGB1-negative nuclei in renal tissue of patients with active AAV was significantly higher than that in normal controls (60.6±20.2% versus 2.7±0.6%, p<0.01). No significant correlation was found between the percentage of HMGB1 negative nuclei in the kidney and histological or clinical findings.

## Discussion

HMGB1, a highly conserved non-histone nuclear protein, acts as a pro-inflammatory mediator extracellularly. There are accumulating evidence that HMGB1 plays an important role in the pathogenesis of RA, IBD and SLE [8–10]. Our previous study found that plasma levels of HMGB1 could reflect the disease activity of AAV [12]. The kidney is one of the most common and potentially life-threatening organs involved in AAV. In the study, we investigated the urinary levels in patients with ANCA-associated glomerulonephritis, and further probed its correlation with clinical and pathological parameters.

In the present study, we demonstrated that the urinary levels of HMGB1 in active AAV patients were significantly higher than AAV patients in remission and healthy controls. Moreover, the level of HMGB1 in remission was lower than that in active phase for most patients with sequential urine samples, which was in line with the study by de Souza et al [22]. Further assessment revealed that the urinary HMGB1 level correlated with ESR, CRP and BVAS, which are commonly used parameters to evaluate the disease activity of AAV. These results indicated that urinary HMGB1, the 30kDa protein, which in blood relates to disease activity, also does so when found in urine. On one hand, HMGB1, as an important proinflammatory mediator, after being released by stimulated neutrophils, macrophages, and monocytes, could stimulate monocytes to secrete a specific subset of proinflammatory cytokines, including TNF-α and IL-1 [7]. On the other hand, HMGB1, as a nuclear protein, can be passively released by mechanically damaged or necrotic cells [23], and HMGB1 in urine may derive from systemic insult, which may account for the lack of association found between urinary HMGB1 levels and the renal histological parameters.

The immunohistochemical staining of HMGB1 in renal biopsies displayed the translocation of HMGB1 from nuclear into cytoplasm. It may reflect active secretion of infiltrated immune cells as well as the injury or necrosis of kidney tissues, which has been shown to arouse necrosis-induced inflammation [24]. The HMGB1-negative nuclei in renal tissue of patients with active AAV nephritis provide one of the explanations for the source of HMGB1 in urine.

There are some limitations of the current study. Firstly, we do not have the urine sample on relapse, since patients were retrospectively recruited. Secondly, the lack of association of histological parameters with urinary HMGB1 limits its utility on reflecting disease activity.

In conclusion, urinary levels of HMGB1 may be associated with the disease activity in AAV patients. It needs to be further explored alongside more meaningful biomarkers. Further studies are needed to investigate whether HMGB1 participates in the pathogenesis of AAV.

### Key messages

Urinary levels of HMGB1 may be associated with the disease activity in AAV patients.
